# Impact of Preformed Donor-Specific Anti-HLA-Cw and Anti-HLA-DP Antibodies on Acute Antibody-Mediated Rejection in Kidney Transplantation

**DOI:** 10.3389/ti.2023.11416

**Published:** 2023-11-22

**Authors:** Timothée Laboux, Rémi Lenain, Jonathan Visentin, Gauthier Flahaut, Paul Chamley, François Provôt, Isabelle Top, Clarisse Kerleau, Myriam Labalette, Gabriel Choukroun, Lionel Couzi, Gilles Blancho, Marc Hazzan, Mehdi Maanaoui

**Affiliations:** ^1^ Department of Nephrology, Kidney Transplantation and Dialysis, CHU Lille, University of Lille, Lille, France; ^2^ INSERM U1167, RID-AGE, University of Lille, Lille, France; ^3^ INSERM UMR 1246 – SPHERE, Nantes University, Tours University, Nantes, France; ^4^ Department of Immunology and Immunogenetics, CHU Bordeaux, Bordeaux, France; ^5^ ImmunoConceEpT, CNRS UMR5164, Inserm ERL U1303, University of Bordeaux, Bordeaux, France; ^6^ Department of Nephrology, Internal Medicine, Dialysis and Transplantation, CHU Amiens, Jules Verne University of Picardie, Amiens, France; ^7^ EA7517, MP3CV Laboratory, Jules Verne University of Picardie, Amiens, France; ^8^ Department of Nephrology, CH Roubaix, Roubaix, France; ^9^ Department of Immunology-HLA, CHU Lille, University of Lille, Lille, France; ^10^ CHU Nantes, Service de Néphrologie-Immunologie Clinique, ITUN, Nantes, France; ^11^ Nantes Université, INSERM, Center for Research in Transplantation and Translational Immunology, UMR 1064, ITUN, Nantes, France; ^12^ INSERM UMR1286, INFINITE, University of Lille, Lille, France; ^13^ Department of Nephrology, Transplantation, Dialysis and Apheresis, CHU Bordeaux, Bordeaux, France; ^14^ INSERM U1190, EGID, Institut Pasteur Lille, CHU Lille, University of Lille, Lille, France

**Keywords:** donor-specific antibodies, kidney transplant, acute antibody-mediated rejection, HLA-Cw, HLA-DP

## Abstract

Given the risk of rejection, the presence of preformed donor specific antibodies (DSA) contraindicates transplantation in most allocation systems. However, HLA-Cw and -DP DSA escape this censorship. We performed a multicentric observational study, in which the objective was to determinate risk factors of acute antibody-mediated rejection (aABMR) in recipients transplanted with preformed isolated Cw- or DP-DSA. Between 2010 and 2019, 183 patients were transplanted with a preformed isolated Cw- or DP-DSA (92 Cw-DSA; 91 DP-DSA). At 2 years, the incidence of aABMR was 12% in the Cw-DSA group, *versus* 28% in the DP-DSA group. Using multivariable Cox regression model, the presence of a preformed DP-DSA was associated with an increased risk of aABMR (HR = 2.32 [1.21–4.45 (*p* = 0.001)]) compared with Cw-DSA. We also observed a significant association between the DSA’s MFI on the day of transplant and the risk of aABMR (HR = 1.09 [1.08–1.18], *p* = 0.032), whatever the DSA was. Interaction term analysis found an increased risk of aABMR in the DP-DSA group compared with Cw-DSA, but only for MFI below 3,000. These results may plead for taking these antibodies into account in the allocation algorithms, in the same way as other DSA.

## Graphical Abstract

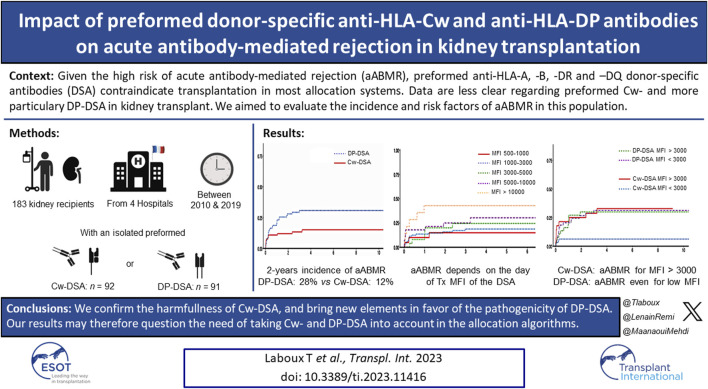

## Introduction

Preformed anti-human leukocyte antigens (HLA) donor-specific antibodies (DSA), especially targeting the A, B, DR and DQ antigens, are reputedly known to be associated with post-transplant rejection [1], with up to 30% of acute antibody-mediated rejection (aABMR) in the first year of transplantation in some series [2–4], and with impaired graft survival [2, 5]. Therefore, most allocation programs introduced the concept of “unacceptable antigens” to avoid kidney transplantation when a preformed DSA is present. However, anti-HLA-Cw and anti-HLA-DP DSA are disregarded in many transplant allocation systems and thus matching algorithms, such as the one provided by the French *Agence de la Biomédecine*, while on the other hand Cw-DSA are mandatory and DP-DSA recommended in the organ allocation system of the United Kingdom for instance [6]. The reasons for this singularity are multiple. First, HLA-Cw and -DP molecules are described as less expressed than other HLA antigens by the endothelial cells [7, 8] and barely immunogenic [9]. Second, the development of bead–based technologies [10] to study anti-HLA antibodies, and more importantly the recent complete and systematic *HLA-C* and *HLA-DP* genotyping of the donor greatly helped to characterize Cw- and DP-DSA. Few clinical cases [11] and retrospective studies [12, 13] have recently provided arguments in favor of a potential pathogenicity of Cw-DSA. However, data are scarce and conflicting regarding isolated preformed DP-DSA. Few small-sized studies did not report any association with aABMR or graft loss with preformed DP-DSA [14, 15], while some cases reported on the contrary hyper-acute ABMR [16, 17]. Some studies also mixed patients with DP- and Cw- DSA, which made their interpretation difficult [18]. As to date, and considering current matching algorithms, the only significant preformed DSA we may face in case of a kidney transplant proposition are Cw- and DP-DSA. The objective of our study was thus to evaluate the incidence of acute ABMR in a multicentric cohort transplanted with either isolated Cw- or DP-DSA, and to identify risk factors of aABMR in this specific population.

## Material and Methods

### Study Patients

The study included every adult transplanted with a single kidney graft in the presence of an isolated preformed Cw- or DP-DSA between 2010 and 2019 at the French University Hospitals of Amiens, Bordeaux, Lille and Nantes. Criteria of exclusion were: pediatric patients, patients presenting another preformed A-, B-, DR- or DQ-DSA, patients presenting with both anti-Cw and anti-DP DSA, ABO-incompatible transplantation, multiorgan transplantation, and patients pre-treated with desensitization protocols before transplantation. Finally, for DP-DSA, as donors’ *HLA-DPA1* genotype was not available for most of the patients, only recipients with at least one anti-DPB1-DSA were included.

### Data Source and Ethical Statement

This multicentric observational study conforms to the tenets of the Istanbul Declaration and the ethical guidelines set forth by the Helsinki Declaration and was approved by the local institutional review boards. No organs were procured from prisoners. All participants provided their informed consent. The dataset was processed under French and European Union data protection laws and regulations (reference: #DEC20-002). This study complies with the “Strengthening the Reporting of Observational Studies in Epidemiology” (STROBE) guidelines [19]. Data from Nantes were collected from the French DIVAT multicentric prospective cohort of kidney and/or pancreatic transplant recipients (www.divat.fr, N°CNIL 914184, ClinicalTrials.gov recording: NCT02900040).

### Data Collection

For each patient, data regarding donor and recipient age, sex, body mass index (BMI), blood group, rank of transplantation, time spent in dialysis, renal-replacement therapy (RRT) method, initial causal nephropathy, calculated panel-reactive antibody (cPRA, defined as the proportion of incompatible grafts that had unacceptable mismatches among proposed deceased kidney donors in the same blood group over the 5 previous years), pre-transplant sensitization in class I and class II antibodies, donor cause of death, cold ischemia time, conservation method, number of *HLA-A*, *-B*, *-DR* and *-DQ* mismatches, induction and peri-transplant prophylactic therapies, and the result of complement-dependent cytotoxicity (CDC) crossmatch, were collected.

### Anti-HLA Antibody Testing

HLA antibodies were detected by single antigen flow beads using a LUMINEX^©^ (LUMINEX 100 or 200) with the LABScreen Single Antigen HLA Class I^©^ and LABScreen Single Antigen HLA Class II^©^ kits (ONE LAMBDA^©^). The antibody level was approximated by the mean fluorescence intensity (MFI). DSA were considered only if present in the serum the day of transplant with a minimum mean fluorescence intensity of 500. In the presence of two or more anti-DP DSA or anti-Cw DSA, the strongest MFI was considered in the analysis. MFI of the preformed Cw- or DP-DSA were secondly monitored at day 15, in the 3rd, 6th, 12th and 24th months post-transplantation.

### Histopathology

The diagnosis of biopsy-proven acute antibody-mediated rejection was performed according to the 2019 Banff classification [20] on “for cause” kidney graft biopsies.

### Endpoints

The primary endpoint was to determine the incidence of aABMR when transplanted with an isolated Cw- or DP-DSA, and then to identify risk factors of aABMR. Secondary endpoints included the identification of risk factors associated with death-censored graft loss and to describe the use of additional prophylactic strategies (Rituximab and/or Intravenous Immunoglobulins (IVIGs) and/or plasmapheresis and/or Eculizumab) performed the day of transplant to prevent rejection in the whole Cw- and/or DP-DSA population.

### Statistical Analysis

The Cw-DSA and the DP-DSA groups were compared on baseline characteristics by chi-2 (categorical data) or Student’s t-tests (continuous data). The Aalen-Johansen estimator was used to estimate event probabilities and to analyze the cumulative incidence of aABMR accounting for the competing risk of death or graft loss for rejection analyses and the competing risk of death for graft loss analysis [21]. Cumulative incidence functions were compared by Gray test when appropriate. Median follow-up times were estimated by a reverse Kaplan Meier method [22]. Hazard ratios for aABMR, and graft loss were computed using Cox proportional hazards modeling. A multivariable backward selection procedure was implemented for the primary endpoint, with a univariate threshold *p* < 0.20 for inclusion and a *p* < 0.05 being defined as statistically significant in the final model. For graft loss, known confounders were included regardless of significance level. An interaction term analysis was performed on the primary endpoint in order to assess the consistency of the effect of MFI on aABMR risk according to the Cw-DSA and the DP-DSA groups. Log-linearity and the proportional hazards assumption were tested using a graphical method. All analyses were carried out in R, version 3.6.3 [23].

## Results

### Study Population and Baseline Characteristics

Among the 183 patients included, 92 were transplanted with isolated preformed Cw- DSA and 91 with preformed DP-DSA. Anti-Cw5 (*n* = 17), anti-Cw7 (*n* = 16) and anti-Cw2 (*n* = 11) were the most frequent Cw-DSA reported, while anti-DP4 (*n* = 21), anti-DP1 (*n* = 13) and anti-DP2 (*n* = 10) were the more frequently found DP-DSA (Supplementary Table S1). The median time of follow-up post-transplant was 4.2 years [Q1: 2.71; Q3: 7.14]. Baseline patients’ characteristics are presented in Tables 1, 2. Overall, mean recipients age was 51.5 years old (±13.0), with a slight over-representation of women. More than half of the patients were retransplanted recipients (51.4%). Mean calculated-PRA was 69.3% (±35.1), with anti-class I and anti-class II HLA sensitization occurring for 89.3% and 76.3% of the patients, respectively. The mean immunodominant DSA MFI at the time of transplantation was 3,540 (±3,537) [Cw-DSA: 3,228 (±3,216); DP-DSA: 3,855 (±3,826), *p* = 0.231]. Of note, four patients were transplanted despite a positive CDC crossmatch (2 Cw-DSA for T-cells and 2 DP-DSA for B-cells). Anti-thymocyte globulins was the main induction therapy (86.4%) and 63 patients (34.4%) were treated with an additional prophylactic protocol the day of transplantation: Rituximab (*n* = 31 [17.3%]), IVIGs (*n* = 45 [25.1%]), plasmapheresis (*n* = 9 [5.03%]), and/or Eculizumab (*n* = 2 [1.12%]). Baseline characteristics were similar between Cw- and DP-DSA recipients, except for BMI [25.4 kg/m^2^ *versus* 23.9 kg/m^2^ respectively (*p* = 0.049)], cPRA [61.5% *versus* 77.3% respectively (*p* = 0.002)], class I HLA sensitization [100% *versus* 77.9% respectively (*p* < 0.001)], class II HLA sensitization [53.5% *versus* 100% respectively (*p* < 0.001)], HLA-DR mismatch [0.84 (±0.72) *versus* 0.54 (±0.62) respectively (*p* = 0.003)], and the use of peri-operative Rituximab [9 (10%) *versus* 22 (24.7%) respectively (*p* = 0.016)].

**TABLE 1:** Baseline characteristics.

	All	Cw-DSA	DP-DSA		
	*n* = 183	*n* = 92	*n* = 91	*p*-value	*n*
Transplant centers				0.633	183
Amiens	8 (4.37%)	5 (5.43%)	3 (3.30%)		
Bordeaux	57 (31.1%)	28 (30.4%)	29 (31.9%)		
Lille	72 (39.3%)	33 (35.9%)	39 (42.9%)		
Nantes	46 (25.1%)	26 (28.3%)	20 (22.0%)		
Recipients					
Age (years)	51.5 (±13.0)	51.7 (±13.4)	51.2 (±12.7)	0.809	183
Sex (% of men)	86 (46.9%)	46 (50.0%)	40 (43.9%)	0.502	183
BMI (kg/m^2^)	24.7 (±4.85)	25.4 (±5.22)	23.9 (±4.34)	0.049	178
Rank of transplantation				0.090	183
1	89 (48.6%)	52 (56.5%)	37 (40.7%)		
2	73 (39.9%)	30 (32.6%)	43 (47.3%)		
3	20 (10.9%)	10 (10.9%)	10 (11.0%)		
5	1 (0.55%)	0 (0.00%)	1 (1.10%)		
ABO blood group				0.488	183
A	84 (45.9%)	47 (51.1%)	37 (40.7%)		
B	18 (9.84%)	8 (8.70%)	10 (11.0%)		
AB	5 (2.73%)	3 (3.26%)	2 (2.20%)		
O	76 (41.5%)	34 (37.0%)	42 (46.2%)		
Time spent in waiting list (days)	1,128 (±1,320)	1,294 (±1,692)	961 (±758)	0.088	183
RRT technique				0.662	183
Preemptive transplant	18 (9.84%)	9 (9.78%)	9 (9.89%)		
Hemodialysis	156 (85.2%)	80 (87.0%)	76 (83.5%)		
Peritoneal dialysis	9 (4.92%)	3 (3.26%)	6 (6.59%)		
Initial nephropathy				0.697	183
Undetermined	21 (11.5%)	11 (12.0%)	10 (11.0%)		
Glomerular	79 (43.2%)	39 (42.4%)	40 (44.0%)		
Vascular	18 (9.84%)	7 (7.61%)	11 (12.1%)		
Tubulo-interstitial	11 (6.01%)	4 (4.35%)	7 (7.69%)		
Polycystic	27 (14.8%)	16 (17.4%)	11 (12.1%)		
Uropathy	27 (14.8%)	15 (16.3%)	12 (13.2%)		
Other organ transplant				0.617	181
Pancreas	4 (2.21%)	1 (1.09%)	3 (3.37%)		
Liver	2 (1.10%)	1 (1.09%)	1 (1.12%)		
Lung	1 (0.55%)	0 (0.00%)	1 (1.12%)		
cPRA (%)	69.3 (±35.1)	61.5 (±37.3)	77.3 (±31.0)	0.002	183
Anti-HLA classe I	159 (89.3%)	92 (100%)	67 (77.9%)	<0.001	178
Anti-HLA classe II	135 (76.3%)	46 (53.5%)	91 (100%)	<0.001	177
Donors					
Age (years)	53.3 (±16.5)	53.2 (±16.4)	53.5 (±16.7)	0.928	183
Sex (% of men)	99 (54.1%)	53 (57.7%)	46 (50.5%)	0.418	183
BMI (kg/m^2^)	26.9 (±5.66)	27.4 (±6.06)	26.3 (±5.18)	0.176	183
Donor type				0.090	183
Vascular brainstem death	95 (51.9%)	53 (57.6%)	42 (46.2%)		
Non-vascular brainstem death	78 (42.6%)	32 (34.8%)	46 (50.5%)		
Living donor	6 (3.28%)	5 (5.43%)	1 (1.10%)		
Maastricht III	4 (2.19%)	2 (2.17%)	2 (2.20%)		
ABO blood group				0.174	183
A	68 (37.2%)	38 (41.3%)	30 (33.0%)		
B	12 (6.56%)	8 (8.70%)	4 (4.40%)		
AB	4 (2.19%)	3 (3.26%)	1 (1.10%)		
O	76 (41.5%)	34 (37.0%)	42 (46.2%)		
Cold ischemia time (min)	1,074 (±490)	1,015 (±536)	1,133 (±435)	0.106	181
Perfusion machine use	58 (31.7%)	32 (34.8%)	26 (28.6%)	0.457	183

BMI, body mass index; cPRA, calculated panel-reactive antibody; RRT, renal replacement therapy.

**TABLE 2:** Histocompatibility and peri-operative prophylactic strategies.

	All	Cw-DSA	DP-DSA		
	*n* = 183	*n* = 92	*n* = 91	*p-*value	*n*
Histocompatibility					
DSA MFI day of transplant	3,540 (±3,537)	3,228 (±3,216)	3,855 (±3,826)	0.231	183
Positive CDC crossmatch	4 (2.19%)	2 (2.17%)	2 (2.20%)	1.000	183
*HLA-A* mismatch number	0.99 (±0.75)	0.95 (±0.69)	1.03 (±0.81)	0.432	183
*HLA-B* mismatch number	1.29 (±0.69)	1.37 (±0.66)	1.21 (±0.72)	0.118	183
*HLA-DQ* mismatch number	0.73 (±0.69)	0.75 (±0.72)	0.72 (±0.65)	0.807	181
*HLA-DR* mismatch number	0.69 (±0.68)	0.84 (±0.72)	0.54 (±0.62)	0.003	183
*A*, *B*, *DR*, *DQ* mismatch number	3.70 (±1.94)	3.91 (±1.91)	3.49 (±1.61)	0.148	181
Induction and desensitization					
Induction therapy				0.787	183
Thymoglobulin	158 (86.3%)	78 (84.8%)	80 (87.9%)		
Anti-CD25	24 (13.1%)	13 (14.1%)	11 (12.1%)		
Alemtuzumab	1 (0.55%)	1 (1.09%)	0 (0.00%)		
Prophylactic treatment					
None	120 (65.6%)	66 (71.7%)	54 (59.3%)	0.108	183
Rituximab	31 (17.3%)	9 (10.0%)	22 (24.7%)	0.016	179
IVIGs	45 (25.1%)	21 (23.3%)	24 (27.0%)	0.698	179
Plasmapheresis	9 (5.03%)	3 (3.33%)	6 (6.74%)	0.330	179
Eculizumab	2 (1.12%)	1 (1.11%)	1 (1.12%)	1.000	179

CDC, complement-dependent cytotoxicity; DSA, donor-specific antibodies; IVIGs, Intravenous Immunoglobulins; MFI, mean fluorescence intensity.

### Primary Endpoint

#### Biopsy-Proven Acute Antibody-Mediated Rejection

**FIGURE 1:**
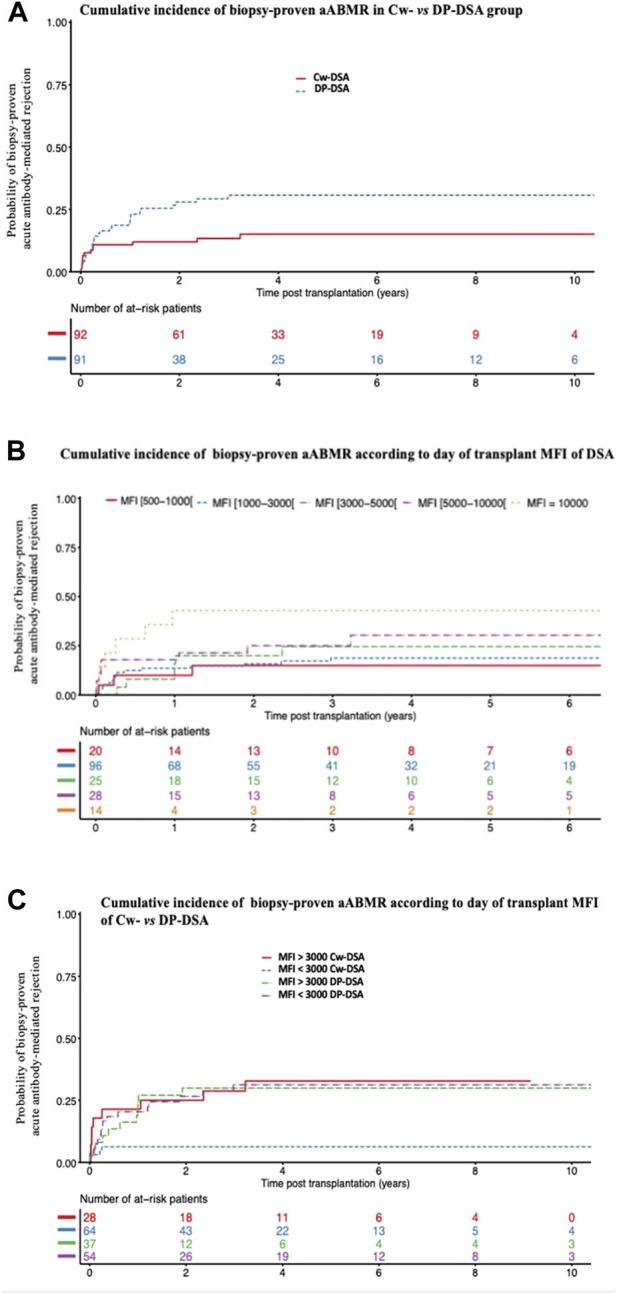
Cumulative incidence of biopsy-proven acute antibody-mediated rejection. **(A)** Cumulative incidence of biopsy-proven aABMR according to Cw- and DP-DSA groups **(B)** Cumulative incidence of biopsy-proven aABMR according to the day of transplant DSA’s MFI **(C)** Cumulative incidence of biopsy-proven aABMR according to the day of transplant MFI of Cw- *versus* DP-DSA. aABMR, acute Antibody-mediated rejection; DSA, Donor Specific Antibody; MFI, Mean Fluorescence Intensity.

During follow-up, 41 of the 183 patients (22.4%) presented a biopsy-proven aABMR including 14 in the Cw-DSA group and 27 in the DP-DSA group. AABMR occurred within a median time of 92 days [Q1: 25; Q3: 370]. Of note, no difference in aABMR prevalence emerged between first and retransplanted-patients (Supplementary Table S2). The 6 months, 1 and 2 years probabilities of aABMR were 10.9% [95% CI 6.0–19.3], 10.9% [95% CI 6.0–19.3] and 12% [95% CI 6.8–20.6] in the Cw-DSA group respectively, *versus* 16.5% [95% CI 10.3–25.8], 22.0% [95% CI 14.8–32.0] and 28% [95% CI 19.8–38.6] in the DP-DSA group, respectively (Figure 1). Multivariable Cox regression showed that preformed DP-DSA were associated with an increased risk of aABMR compared with Cw-DSA, with an adjusted Hazard Ratio (aHR) of 2.25 [1.17–4.31] (*p* = 0.015) (Table 3). Regardless of the nature of the preformed DSA, day of transplant MFI was independently associated with the risk of aABMR, with an aHR of 1.09 [1.08–1.18] (*p* = 0.032) per 1000 MFI increment (Table 3). Other variables associated with the risk of aABMR were recipient age [aHR = 0.76 [0.60–0.97] (*p* = 0.026)] and a positive CDC crossmatch the day of transplant [aHR = 4.59 [1.03–20.38] (*p* = 0.045)]. For MFI below 3,000, the risk for aABMR was increased in the DP-DSA group compared with Cw-DSA group, with an aHR of 4.69 [1.68–13.08]. Conversely, there was no significant difference between the groups for MFI greater than 3,000 (aHR 1.05 [0.43–2.57]), suggesting that the increased risk observed of aABMR in the DP-DSA group compared with Cw-DSA mostly concerned DSA with MFI < 3,000 (Table 4).

**TABLE 3:** Multivariable Cox model for the risk of biopsy-proven acute antibody-mediated rejection.

	Biopsy-proven acute ABMR	
	Multivariate	*p*-value
	HR [95% CI]	
Preformed DP- *vs* Cw-DSA	2.25 [1.17–4.31]	0.015
Day of transplant DSA’s MFI (/1,000 MFI increment)	1.09 [1.01–1.18]	0.032
Recipient age (per 10 years)	0.76 [0.60–0.97]	0.026
CDC crossmatch positivity (vs. negativity)	4.59 [1.03–20.4]	0.045

ABMR, antibody-mediated rejection; CDC, complement-dependent cytotoxicity; DSA, donor-specific antibodies; MFI, mean fluorescence intensity.

**TABLE 4:** Multivariate interaction term model for the risk of biopsy-proven acute antibody-mediated rejection.

	Biopsy-proven acute ABMR	
	Multivariate	*p*-value*
	HR [95% CI]	
Preformed DP- *vs.* Cw-DSA/MFI < 3,000	4.69 [1.68–13.1]	0.033
Preformed DP- *vs.* Cw-DSA/MFI > 3,000	1.05 [0.43–2.57]	

This model was adjusted for recipient age and CDC crossmatch positivity. ABMR, antibody-mediated rejection; DSA, donor-specific antibodies; MFI, mean fluorescence intensity*.*

* The calculated *p*-value stands for the whole interaction term multivariate analysis.

#### Post-Transplant DSA Monitoring

To ensure the plausibility of the effect of preformed Cw- and/or DP-DSA on the occurrence of aABMR, we monitored the post-transplant evolution overtime of the preformed DSA’s MFI. In patients who experienced aABMR, mean MFI decreased from 4,446 (±3,898 SD) at the day of transplant, to 4,175 (±4,729 SD) at day 15, 2,916 (±4,934 SD) at 3 months, 2,487 (±4,191 SD) at 6 months, 1,758 (±3,139 SD) at 12 months and finally to 1,506 (±3,295 SD) at 24 months. However, strikingly, mean MFI of the preformed DSA was still at 4,463 (±5,257 SD) on the onset of aABMR. In patients who did not experience aABMR, mean MFI decreased as well from 3,191 (±3,239 SD) at the day of transplant, to 2,707 (±3,652 SD) at day 15, 1,698 (±2,589 SD) at 3 months, 1,866 (±2,424 SD) at 6 months, 1,350 (±2,324 SD) at 12 months and finally to 1,127 (±2,187 SD) at 24 months. Mean DSA’s MFI follow-up is presented in Supplementary Figure S1. Thirty-eight *de novo* DSA (*dn*DSA) appeared during the follow-up in 25 out of the 183 (13.7%) patients (13 in the Cw-DSA group, and 12 in the DP-DSA group). The median time to onset of *dn*DSA was 494 days [Q1: 101; Q3: 882]. *De novo* DSA were directed against the *loci* A (*n* = 3), B (*n* = 10), Cw (*n* = 5), DR (*n* = 11), DQ (*n* = 5), and DP (*n* = 4). Ten of these 25 patients will present aABMR during follow-up. The onset of *dn*DSA appeared to be attributable to aABMR in 6 of these 10 aABMR-patients (14.4% of the whole aABMRs observed here), with a concomitant or shortly appearance of *dn*DSA preceding the acute rejection episode (two patients in the Cw-DSA group, and four patients in the DP-DSA group). For the other four patients, the *dn*DSA appeared largely after the occurrence of aABMR (median time between rejection and the onset of *dn*DSA (in this order): 1860 days [Q1: 481; Q3: 2,701]), and were therefore considered as unrelated to the development of aABMR (Supplementary Table S3). Taken together, *dn*DSA emergence may therefore interfere here with 14.4% of the aABMR onset, letting the 85.6% other aABMR+ patients with no other DSA than the preformed Cw- or DP-DSA.

### Secondary Endpoints

#### Graft Loss

**FIGURE 2:**
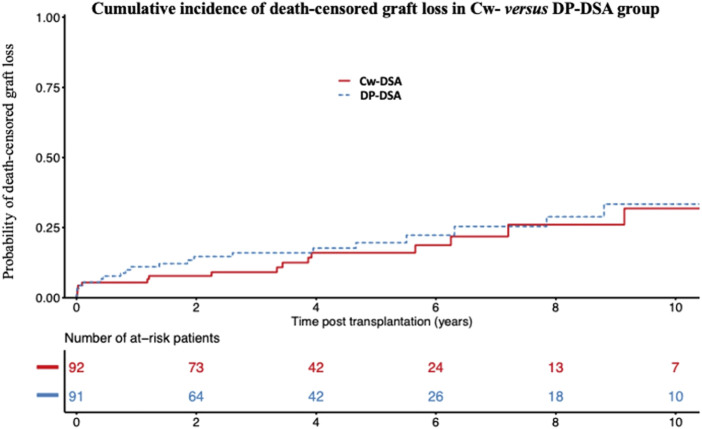
Cumulative incidence of death-censored graft loss. Cumulative incidence of death-censored graft loss in the Cw- *versus* DP-DSA group. DSA, Donor Specific Antibody.

Considering graft loss, death-censored graft loss occurred for 41 of the 183 patients (Cw-DSA: 18; DP-DSA: 23). The median time until death-censored graft loss was 2.3 years [Q1: 0.4; Q3: 6.3]. Probabilities of death-censored graft loss at 1, 3, and 5 years were 5.4% [95% CI 2.3–12.6], 9.1% [95% CI 4.7–17.4] and 16.0% [95% CI 9.2–26.8] in the Cw-DSA group *versus* 11.0% [95% CI 6.1–19.5], 15.9% [95% CI 9.7–25.5] and 19.5% [95% CI 12.3–30.3] in the DP-DSA group (Figure 2), respectively. Multivariable Cox regression model did not find any significant association between the type of DSA or the level of MFI with death-censored graft loss (Table 5).

**TABLE 5:** Multivariable Cox model for the risk of development of death-censored graft loss.

	Death-censored graft loss	
	Multivariate	*p*-value
	HR [95% CI]	
Preformed DP- vs. Cw-DSA	1.10 [0.55–2.23]	0.786
Day of transplant DSA’s MFI (/1,000 MFI increment)	1.04 [0.95–1.14]	0.358
Recipient age (/10 years)	0.87 [0.65–1.17]	0.368
Recipient sex (male vs. female)	1.27 [0.60–2.69]	0.532
Recipient BMI (per 1 kg/m^2^)	1.06 [0.98–1.15]	0.127
Rank of transplantation (one vs. several)	1.90 [0.75–4.80]	0.174
Waiting time on list (per day)	1.00 [1.00–1.00]	0.411
Cold ischemia time (per minute)	1.00 [1.00–1.00]	0.202

BMI, body mass index; DSA, donor-specific antibodies; MFI, mean fluorescence intensity.

#### Additional Prophylactic Treatment the Day of Transplant

**FIGURE 3:**
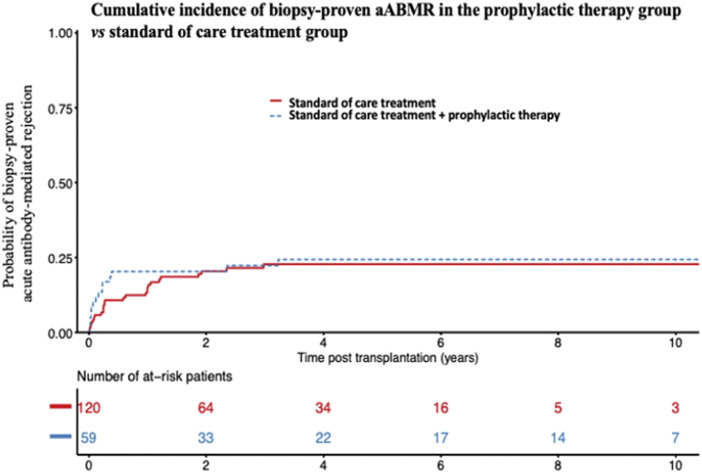
Cumulative incidence of biopsy-proven acute ABMR after supplemental prophylactic therapy on the day of transplant. Cumulative incidence of biopsy-proven aABMR in the prophylactic therapy-group (Rituximab and/or IVIGs and/or plasmapheresis and/or Eculizumab) *versus* standard of care alone-treated group. aABMR, acute Antibody-mediated rejection; IVIGs, Intravenous Immunoglobulins.

A total of 63 patients (34.4%) were treated with an additional prophylactic treatment on the day of transplant, in addition to conventional induction, as previously described. None of the treatments had any significant effect in univariate analyses (Figure 3). Overall, among the 63 patients who received any additional prophylactic therapy on the day of transplant (Rituximab and/or IVIGs and/or plasmapheresis and/or Eculizumab), 15 experienced aABMR (23.8%), *versus* 26 out of the 120 patients who received the standard of care treatment (21.6%) (*p* = 0.65). AABMR occurred in 9 out of 31 patients treated with Rituximab (29%), *versus* 32 out of 131 other patients who were not (24.4%). Twelve out of 45 patients treated by IVIGs (26.6%) experienced aABMR, *versus* 29 out of 134 other patients who were not (21.6%). Two of the 9 patients treated by plasmapheresis (22.2%), compared with 39 out of 170 (22.9%) presented with aABMR. None of the two patients who received Eculizumab experienced rejection.

## Discussion

In this study, we described the incidence and risk factors associated with aABMR in a multicentric observational study of recipients transplanted in the presence of isolated preformed Cw- or DP-DSA. Two years after transplantation, the probability of developing an aABMR was 12% and 28% for patients transplanted with a preformed Cw- or DP-DSA, respectively. In multivariate analysis, the presence of a preformed DP-DSA was associated with approximately twice the risk of aABMR compared with Cw-DSA. We also found that the MFI of the DSA at the time of transplantation was significantly associated with aABMR, whatever the DSA was, and that there was a significant interaction between the nature of the DSA and the MFI. The increased risk associated with DP-DSA, compared with Cw-DSA, was significant only for MFI below 3,000. No difference was found between the groups in terms of death-censored graft loss. Finally, the use of a prophylactic therapy the day of transplantation to prevent rejection did not seem to be associated with a lower incidence of aABMR.

Twelve percent of patients transplanted in the presence of a Cw-DSA presented in our study an aABMR at 2 years of follow-up. This incidence is lower than those reported in previous reports for Cw-DSA, between 20% and 30% [13, 24]. These results may have been impacted by a non-estimated proportion of denatured anti-HLA-Cw. Like all class I molecules, HLA-Cw can lose its β2m-chain, leading to the denaturation of the HLA molecule. Sensitization against cryptic antigens of these denatured class I molecules is frequent, yet clinically irrelevant [25]. Compared with anti-HLA-A and -B, denatured anti-HLA-Cw are particularly prevalent, corresponding to 10% of antibodies from pre-transplant patients and up to 40% of DSA in sensitized kidney transplant recipients [24, 26]. Using acid-treated Luminex beads (iBeads^®^, One Lambda) recognizing only native class I anti-HLA, Visentin et al., showed a prevalence of nearly 45% of denatured anti-HLA-Cw (23 of 52 patients with isolated preformed Cw-DSA). The authors revealed then a 2 years incidence of aABMR of 55% (16/29 patients) in the native Cw-DSA group, compared with 8.7% (2/23 patients) in the denatured Cw-DSA group (*p* = 0.006). This increase in aABMR was clinically reflected by a significant decrease in graft survival in the native Cw-DSA group [12]. Interestingly, in this study, mean baseline MFI of native Cw-DSA were significantly and importantly higher than of denatured anti-HLA-Cw antibodies (5,503 [1,655–8,198] *versus* 998 [742–2,140]) [12]. We may assume then that in our population, denatured DSA may be present in the lowest categories of Cw-DSA MFI, which would explain the difference of risk of aABMR associated with Cw-DSA below and over the 3,000 threshold. Considering that nowadays the probability of being transplanted in the presence of a preformed DSA is almost exclusively limited to Cw- and DP-DSA, the challenge in this population remains therefore to successfully identify pathogenic Cw-DSA, in order to help further stratify the risk. In addition to iBeads mentioned above [12], other tools such as the ability of the DSA to bind C1q [27], C3d [28], or the identification of DSA’s IgG subclass [29] could be useful, and deserve to be tested specifically in this population.

We report here the largest cohort to our knowledge of patients transplanted in the presence of an isolated preformed DP-DSA, confirming the alleged association of these antibodies with aABMR. The pathogenicity of DP-DSA has indeed been raised by several case-reports [16, 17, 30–33] and clearly suggested by Bachelet et al., which provided a pooled analysis of Cw- and DP-DSA preformed DSA [18]. We report here a 2 years-incidence of aABMR of 28% in the presence of a preformed DP-DSA at the time of kidney transplantation. This is consistent with the recent report from the Swiss transplant cohort study, who also found a 2 years prevalence of aABMR of around 25% in 33 recipients transplanted in the presence of an isolated preformed DP-DSA, results of note no different from those observed in DR- or DQ-DSA patients in this same study [34]. The nature of the rejection (aABMR), the persistence of significant post-transplant MFI of the preformed DP-DSA at the time of aABMR [3,178 (±4,618 SD)] despite natural post-transplant decrease, the relatively low proportion of *de novo* DSA appearance who could have interfered with the onset of aABMR [only 4 out of 27 (14.8%) aABMR attributable to a *dn*DSA emergence in the DP-DSA group], and finally the significant association between the MFI of the preformed DP-DSA and aABMR, are all together strong arguments in favor of the pathogenicity of these antibodies. Despite quite early onset of aABMR, anamnestic B-cell response did not seem to be the main immunological pathway here, as DSA’s MFI did not strongly increase at day 15, and as no difference was observed here between first and retransplanted-patients. Noteworthily, distribution of antigenic specificities of DP-DSA matched here with the prevalence of the different *HLA-DPB1* alleles expressed by the general populations [35]. In our cohort of 183 sensitized patients transplanted with a preformed DSA, the presence of a DP-DSA was associated with a two-fold increased risk compared with Cw-DSA. Using interaction analyses, we also showed that the risk was dependent of the DSA’s MFI. Below an MFI of 3,000, DP-DSA had approximately a 4-fold increased risk compared with Cw-DSA, but this risk disappeared for MFI over 3,000. Taken together, these results could suggest that the Cw-DSA pathogenicity would be proportional to its MFI on the day of transplantation for values greater than 3,000, whereas the pathogenic effect of DP-DSA would be constant, and would appear whatever the MFI is. Conversingly, the recent Swiss transplant cohort study already discussed above found similar results for all classe II-DSA, including DP-DSA, demonstrating an association with aABMR and death-censored graft loss even for MFI < 1,000, while classe I-DSA (including 28 Cw-DSA patients) where associated with aABMR only for MFI > 1,000 [34]. In a historical cohort, Lefaucheur et al., demonstrated a prevalence of aABMR in a sensitized cohort of patients without DSA of only 0.94% (3/319 patients) at 8 years, a result largely below the 28% of aABMR observed in our study at 2 years in the DP-DSA group [2]. The prevalence of aABMR in this same study was conversely 34.9% (29/83 patients) in the group transplanted in the presence of a preformed anti-A, -B, -DR or -DQ DSA [2], results close to those observed in our study in the DP-DSA group. Taken together, these results suggest that DP-DSA may exhibit a pathogenicity at least similar to other DSA included in “unacceptable antigens” allocation programs.

Finally, our study did not show any trend in favor of a reduction of aABMR after peri-operative prophylactic treatment to prevent rejection. In a small prospective cohort, Akalin et al., showed a mean decrease in the MFI of preformed DSA in the group treated with IVIGs and plasmapheresis of 38% (*n* = 14), compared with a decrease of 24% in the group receiving only IVIGs (*n* = 9). The prevalence of aABMR was 44% (4 patients out of 9) in the IVIGs alone group *versus* 7% (1 patient out of 14) in the IVIGs + plasmapheresis group [36]. In a prospective uncontrolled study, Jin et al., reported no episode of acute rejection in 7 HLA-incompatible transplant-recipients (presence of a DSA on transplant day) treated with peri-operative low-dose IVIGs, plasmapheresis and Rituximab over a mean follow-up of 3 years [37]. Finally, in a retrospective cohort of 50 sensitized recipients transplanted in the presence of a preformed DSA, treated (*n* = 25) or not treated (*n* = 25) with Rituximab in addition to treatment with IVIGs and peri-operative plasmapheresis, the Rituximab-treated patients had less DSA rebound during follow-up. However, there was a similar proportion of biopsy-proven acute rejection and especially aABMR (4 *versus* 6, *p* = 0.23), with similar graft survival between the two groups [38]. It is important to emphasize that none of these studies, retrospective and/or with small number of patients, included Cw- or DP-DSA. Although no trend in favor of an aggressive prophylactic strategy emerged from our observation, our retrospective study was not designed to evaluate the efficacy of such therapies. Further randomized studies would be warranted then to assess the validity of such treatments.

Our findings need to be interpreted in the context of some caveats. First, the retrospective nature of the study could be associated with information bias. Another limitation pertains to the absence of a control group without Cw- or DP-DSA. Including a control group, while essential for drawing meaningful conclusions, poses challenges related to potential biases that could compromise the validity of our findings. To navigate this issue, we deliberately refrained from introducing such a control group. On the one hand, a control group comprising exclusively non-sensitized recipients would not permit us to distinguish between the impact of HLA sensitization and the effect of preformed DSA. HLA sensitization is recognized to influence the likelihood of acute rejection and graft loss, even in the absence of DSA [39]. Therefore, introducing this group would confound our ability to isolate the specific effects of DSA. On the other hand, forming a control group solely consisting of sensitized recipients without preformed DSA introduces biases associated with prevailing definitions of HLA sensitization in allocation systems. In France, as in many other countries, HLA sensitization is assessed using the cPRA, which is contingent upon the prevalence of HLA antigens within the allocation population. Comparing outcomes between sensitized recipients with equivalent cPRA values assumes uniform levels of sensitization, disregarding the nuanced nature of HLA antibodies according to the prevalence of HLA antigens in the French population. Considering the current debate surrounding cPRA’s effectiveness in stratifying immune risk [40], we opted to restrict our analyses to a specific population in the French allocation system that can be transplanted with preformed DSA—namely, those with Cw- or DP-specific DSA. This approach allows us to more directly assess the impact of these specific DSA while minimizing potential biases inherent in broader control groups.

In conclusion, the 2 years incidence of acute ABMR in this multicentric study was 12% and 28% for patients transplanted in the presence of a preformed Cw- or DP-DSA, respectively. The pathogenicity of Cw-DSA was MFI-dependent, and appeared essentially for MFI superior to 3,000, while the increased risk of aABMR occurred even for low-MFI value DP-DSA. Taken together, these results suggest that Cw- and DP-DSA might present a pathogenicity at least equivalent to other DSA included in “unacceptable antigens” program. Today, no consensual attitude exists in most allocation systems regarding Cw-DSA and DP-DSA. Our results may therefore question the need of taking these antibodies into account in the allocation algorithm, in the same way as the other anti-HLA antibodies.

## Data Availability

The raw data supporting the conclusion of this article will be made available by the authors, without undue reservation.

## Abbreviations

95%CI, 95% confidence interval; aABMR, acute antibody-mediated rejection; ABMR, antibody-mediated rejection; aHR, adjusted hazard ratio; BMI, body mass index; CDC, complement-dependent cytotoxicity; cPRA, calculated panel-reactive antibody; DSA, donor specific antibody; HLA, human leukocyte antigens; HR, hazard ratio; IVIGs, intraveinous immunoglobulins; MFI, mean fluorescence intensity; PRA, panel-reactive antibody; Q1, first quartile; Q3, third quartile; RRT, renal replacement therapy.
