# Supplementary material for: Impact of Preformed Donor-Specific Anti-HLA-Cw and Anti-HLA-DP Antibodies on Acute Antibody-Mediated Rejection in Kidney Transplantation
Source: Transpl Int. 2023 Nov 22;36:11416. doi: 10.3389/ti.2023.11416 (PMC10698113; doi:10.3389/ti.2023.11416)

SUPPLEMENTARY MATERIALS

Supplemental Table S1.

Distribution of antigenic specificities and proportion of aABMR of Cw- and DP-DSA patients, put in parallel with the distribution of the top 4 most frequent *HLA-C* and *HLA-DPB1* alleles shared by the Creary LE *et al. European American cohort (*n* = 2248).**

***aABMR**: acute antibody-mediated rejection ; **DSA**: donor-specific antibodies.*

***** Creary LE, Gangavarapu S, Mallempati KC *et al.* Next-generation sequencing reveals new information about *HLA* allele and haplotype diversity in a large European American population. *Hum Immunol.* 2019;80(10): 807-822

Supplemental Table S2.

Prevalence of acute ABMR in first *versus* retransplanted-patients

***aABMR**: acute antibody-mediated rejection ; **DSA**: donor-specific antibodies.*

Supplemental Table S3.

***De novo* DSA appearance and acute antibody-mediated rejection**

aABMR**: acute antibody-mediated rejection ;* **dn*DSA: de novo *donor-specific antibodies.*

Supplemental Figure S1.

Post-transplant monitoring of preformed Cw- or DP-DSA's MFI.

A) Post-transplant MFI of preformed Cw- or DP-DSA in patients who experienced aABMR. **B)** Post-transplant MFI of preformed Cw- or DP-DSA in aABMR-free patients.

***aABMR**: acute Antibody-mediated rejection ; **D15**: day 15 post-transplant ; **DOT**: day of transplant ; **DSA**: donor specific antibody ; **Mx**: mounth x post-transplant; **MFI**: Mean Fluorescence Intensity.*

Supplemental Table S1. Distribution of antigenic specificities and proportion of aABMR of Cw- and DP-DSA patients, put in parallel with the distribution of the top 4 most frequent *HLA-C* and *HLA-DPB1* alleles shared by the Creary LE *et al.** European American cohort (*n* = 2248).

Cw-DSA	*n*	aABMR	DP-DSA	*n*	aABMR
**Anti-Cw5**	**17**	3 (17.6%)	Anti-DP1	13	4 (30.7%)
**Anti-Cw7**	**16**	2 (12.5%)	**Anti-DP402**	**12**	2 (16.7%)
**Anti-Cw2**	**12**	3 (25.0%)	**Anti-DP2**	**10**	3 (33.3%)
Anti-Cw4	10	0 (0.00%)	**Anti-DP401**	**9**	1 (11.1%)
Anti-Cw12	10	1 (10.0%)	**Anti-DP3**	**8**	3 (37.5%)
Anti-Cw6	9	2 (22.2%)	Anti-DP5	8	2 (25.0%)
Anti-Cw9	4	0 (0.00%)	Anti-DP11	7	1 (14.3%)
Anti-Cw10	4	0 (0.00%)	Anti-DP14	6	5 (83.3%)
Anti-Cw1	3	1 (33.3%)	Anti-DP6	5	0 (0.00%)
Anti-Cw16	2	1 (50.0%)	Anti-DP13	3	3 (100%)
**Anti-Cw3**	**1**	0 (0.00%)	Anti-DP17	3	1 (33.3%)
Anti-Cw8	1	0 (0.00%)	Anti-DP9	2	1 (50.0%)
Anti-Cw14	1	0 (0.00%)	Anti-DP10	2	0 (0.00%)
Anti-Cw15	1	1 (100%)	Anti-DP15	1	0 (0.00%)
Anti-Cw17	1	0 (0.00%)	Anti-DP19	1	0 (0.00%)
			Anti-DP20	1	1 (100%)

Top 4 *HLA-C* expression*

1. *HLA-C*07* (26.8%)
2. *HLA-C*03* (12.9%)
3. *HLA-C*05* (7.9%)
4. *HLA-C*02* (4.4%)

Top 4 *HLA-DPB1* expression*

1. *HLA-DPB1*04:01* (42.6%)
2. *HLA-DPB1*02* (13.8%)
3. *HLA-DPB1*04:02* (11.6%)
4. *HLA-DPB1*03.* (8.4%)

***aABMR**: acute antibody-mediated rejection ; **DSA**: donor-specific antibodies.*

***** Creary LE, Gangavarapu S, Mallempati KC *et al.* Next-generation sequencing reveals new information about *HLA* allele and haplotype diversity in a large European American population. *Hum Immunol.* 2019;80(10): 807-822

Supplemental Table S2. Prevalence of acute ABMR in first *versus* retransplanted-patients

	**All** *n = 183*	**Cw-DSA** *n = 92*	**DP-DSA** *n = 91*	*n*
aABMR in the whole cohort	41 (22.4%)	14 (15.2%)	27 (29.7%)	183
aABMR in 1st transplanted-patients	17 (19.1%)	6 (11.5%)	11 (29.7%)	89
aABMR in retransplanted-patients	24 (25.5%)	8 (20.0%)	16 (29.6%)	94

aABMR: *acute antibody-mediated rejection ;* ***DSA***: *donor-specific antibodies.*

Supplemental Table S3. *De novo* DSA appearance and acute antibody-mediated rejection

	**All** *n = 183*	**Cw-DSA** *n = 92*	**DP-DSA** *n = 91*	*n*
***De novo* DSA onset**	25 (13.7%)	13 (14.1%)	12 (13.2%)	183
aABMR	41 (22.4%)	14 (15.2%)	27 (29.7%)	183
*dn*DSA + aABMR	10 (24.3%)	5 (35.7%)	5 (18.5%)	41
*dn*DSA preceding aABMR	6 (14.6%)	2 (14.3%)	4 (14.8%)	41

aABMR*: acute antibody-mediated rejection ;* **dn*DSA****:* de novo *donor-specific antibodies.*

Supplemental Figure S1

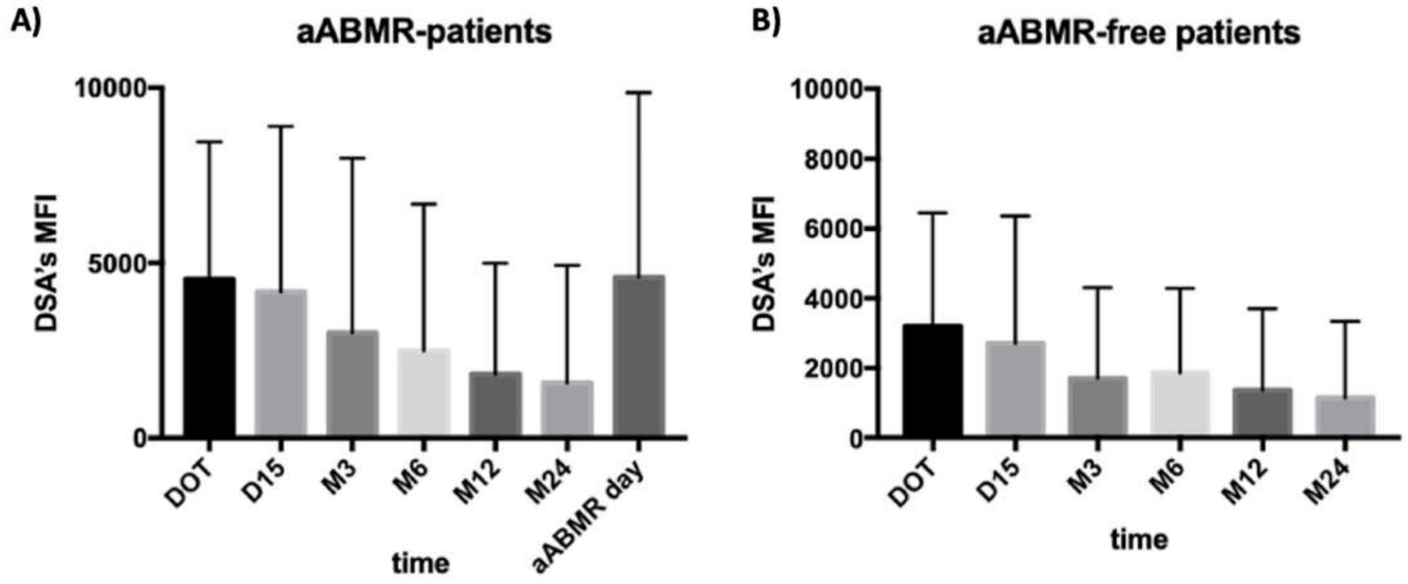

Supplement: Supplementary file 1 [file DataSheet1.pdf]
